# Expanding the utility of the ROX index among patients with acute hypoxemic respiratory failure

**DOI:** 10.1371/journal.pone.0261234

**Published:** 2022-04-26

**Authors:** Andrew Li, Matthew Edward Cove, Jason Phua, Ser Hon Puah, Vicky Ng, Amit Kansal, Qiao Li Tan, Juliet Tolentino Sahagun, Juvel Taculod, Addy Yong-Hui Tan, Amartya Mukhopadhyay, Chee Kiang Tay, Kollengode Ramanathan, Yew Woon Chia, Duu Wen Sewa, Meiying Chew, Sennen J. W. Lew, Shirley Goh, Shekhar Dhanvijay, Jonathan Jit-Ern Tan, Kay Choong See FCCP, on behalf of SICM-NICER Group

**Affiliations:** 1 Division of Respiratory and Critical Care Medicine, Department of Medicine, National University Hospital, National University Health System, Singapore, Singapore; 2 Department of Intensive Care Medicine, Woodlands Health, Singapore, Singapore; 3 Fast and Chronic Programmes, Alexandra Hospital, National University Health System, Singapore, Singapore; 4 Department of Respiratory and Critical Care Medicine, Tan Tock Seng Hospital, Singapore, Singapore; 5 Department of Anaesthesiology, Intensive Care and Pain Medicine, Tan Tock Seng Hospital, Singapore, Singapore; 6 Department of Intensive Care Medicine, Ng Teng Fong General Hospital, National University Health System, Singapore, Singapore; 7 Department of Respiratory Medicine and Critical Care Medicine, Singapore General Hospital, Singapore, Singapore; 8 Division of Critical Care, National University Hospital, National University Health System, Singapore, Singapore; 9 Department of Anaesthesia, National University Hospital, National University Health System, Singapore, Singapore; 10 Department of Cardiac Thoracic and Vascular Surgery, National University Heart Centre, National University Hospital, Singapore, Singapore; 11 Department of Cardiology, Tan Tock Seng Hospital, Singapore, Singapore; Kaohsuing Medical University Hospital, TAIWAN

## Abstract

**Background:**

Delaying intubation in patients who fail high-flow nasal cannula (HFNC) may result in increased mortality. The ROX index has been validated to predict HFNC failure among pneumonia patients with acute hypoxemic respiratory failure (AHRF), but little information is available for non-pneumonia causes. In this study, we validate the ROX index among AHRF patients due to both pneumonia or non-pneumonia causes, focusing on early prediction.

**Methods:**

This was a retrospective observational study in eight Singapore intensive care units from 1 January 2015 to 30 September 2017. All patients >18 years who were treated with HFNC for AHRF were eligible and recruited. Clinical parameters and arterial blood gas values at HFNC initiation and one hour were recorded. HFNC failure was defined as requiring intubation post-HFNC initiation.

**Results:**

HFNC was used in 483 patients with 185 (38.3%) failing HFNC. Among pneumonia patients, the ROX index was most discriminatory in pneumonia patients one hour after HFNC initiation [AUC 0.71 (95% CI 0.64–0.79)], with a threshold value of <6.06 at one hour predicting HFNC failure (sensitivity 51%, specificity 80%, positive predictive value 61%, negative predictive value 73%). The discriminatory power remained moderate among pneumonia patients upon HFNC initiation [AUC 0.65 (95% CI 0.57–0.72)], non-pneumonia patients at HFNC initiation [AUC 0.62 (95% CI 0.55–0.69)] and one hour later [AUC 0.63 (95% CI 0.56–0.70)].

**Conclusion:**

The ROX index demonstrated moderate discriminatory power among patients with either pneumonia or non-pneumonia-related AHRF at HFNC initiation and one hour later.

## Introduction

High-flow nasal cannula therapy (HFNC) provides many benefits to critically ill patients, including reduced work of breathing, reliable oxygen delivery at higher concentrations and enhanced secretion clearance [1–3]. HFNC has gained popularity [4–6] since the landmark FLORALI study for acute hypoxemic respiratory failure (AHRF) among patients with pneumonia [7]. Many centres have broadened indications beyond pneumonia [8,9], using it in pulmonary hemorrhage [10], acute exacerbation of chronic obstructive pulmonary disease (COPD) [9,11–13] and heart failure [14]. Its use has extended beyond the intensive care unit (ICU) to the emergency department (ED), where the aetiology of the hypoxemic respiratory failure may not yet be ascertained [9,14].

Although HFNC has been used among AHRF patients with non-pneumonia indications, existing evidence for HFNC use has been mainly among pneumonia patients. The FLORALI study demonstrated lower intubation rates among pneumonia patients with PaO_2_:FiO_2_ ratio (PF ratio) less than 200 mmHg when compared to those receiving conventional oxygen therapy (COT) and non-invasive ventilation (NIV), although up to 40% still required intubation [7]. Prior studies among non-pneumonia patients focused mainly on physiological parameters [8–14], with the failure rate remaining largely unknown. Understanding HFNC failure rates is important, because mortality may be increased if intubation is delayed [15].

In this respect, Roca and colleagues developed the ROX index to help predict HFNC failure in patients with pneumonia [16,17]. The ROX index is the ratio of SpO_2_/FiO_2_ (SF ratio) to respiratory rate. It has excellent specificity (98–99%) for predicting HFNC failure at various time points of 2 (<2.85), 6 (<3.47) and 12 hours (<3.85), albeit at the expense of low sensitivity [17]. Physicians have subsequently attempted to improve the ROX index [18] and explore its utility as a predictor of weaning failure [19]. Current evidence supporting the use of the ROX index is limited to pneumonia patients mainly at later time points, limiting its utility.

Understanding the performance of the ROX index at earlier time points, would enhance prediction of HFNC failure, facilitating earlier intubation and potentially averting increased mortality risks. In this study, we aimed to expand the utility of the ROX index as a tool for predicting HFNC failure by validating its use at earlier time points among both pneumonia and non-pneumonia patients. We hypothesized that the ROX index could discriminate patients likely to experience HFNC failure at earlier time points in both pneumonia and non-pneumonia AHRF.

## Methods

### Study design

We conducted a retrospective observational multi-center study in all public hospital intensive care units (ICU) in Singapore of patients using HFNC for AHRF from 1 January 2015 to 30 September 2017. The National Healthcare Group Domain-Specific Review Board approved the study with a waiver of informed consent due to the non-interventional study design (DSRB 2017/00900).

### Patients

Patients were included if they were older than 18 years and treated with HFNC for AHRF. Patients with concomitant hypercapnia were also included. Patients were excluded if they had do-not-intubate orders for the current AHRF episode or if HFNC was used for pre-oxygenation, post-extubation management or peri-procedural purposes. HFNC initiation was at the discretion of the primary physician.

### Device description and management

HFNC was provided with one of the following devices: Optiflow device (MR850 heated humidified delivery tubing and nasal cannula; Fisher & Paykel Healthcare, Auckland, New Zealand)^™^, Bio-med device high flow air-oxygen blender with the heated humidifier (MR850, Fisher & Paykel Healthcare, Auckland, New Zealand)^™^ or the Airvo 2 device (Fisher & Paykel Healthcare, Auckland, New Zealand)^™^. Despite the different devices, all the nasal cannula interfaces were similar to the Airvo2 device, where the nasal cannula is able to deliver humidified respiratory gases up to flows of 70L/min. Physicians initiated HFNC at a minimum flow of 30L/min with FiO_2_ of at least 30% to target a SpO_2_ of at least 92%.

### Data collection

Demographic characteristics were collected for all patients including: age, gender, ethnicity, admission source and comorbidities (diabetes, hypertension, ischemic heart disease, stroke, asthma, COPD, bronchiectasis, interstitial lung disease, liver cirrhosis and chronic kidney disease). Immunocompromised state was also determined, defined as the presence of any solid tumor, hematological malignancy, or usage of steroids (at least 0.3mg/kg/day for at least 1 month) [20] or other immunosuppressants. Illness severity was determined from the Acute Physiology and Chronic Health Evaluation (APACHE) II score and frequency of vasopressor use.

The underlying etiology of AHRF was categorized into one of the following: (a) pneumonia, defined as the presence of respiratory symptoms with radiological evidence on chest radiograph; (b) heart failure; (c) COPD exacerbation; (d) asthma exacerbation; (e) exacerbation of interstitial lung disease; (f) HFNC use post-surgery; (g) others. In cases where multiple etiologies may account for the patient’s AHRF, the leading etiology was determined by the primary physician.

HFNC failure was defined as requiring intubation after HFNC initiation. However, since this was a retrospective observational study, and none of the participating ICUs used a specific protocol to determine intubation decisions among the HFNC patients, the intubation decision occurred at the discretion of the managing physician. ICU mortality, hospital mortality, ICU length-of-stay and hospital length-of-stay were also collected.

As mentioned, the ROX index is the ratio of the SF ratio to respiratory rate. In order to compute the ROX index as defined by Roca and colleagues [17], the following parameters were collected immediately prior to HFNC initiation, after one hour and in the event of HFNC failure, at the point of intubation: SpO_2_ (%), FiO_2_ (%), respiratory rate (breaths/min), flow rate (L/min) and arterial partial pressure of carbon dioxide (PaCO_2_, in mmHg) [16,17].

### Statistical analysis

Univariate comparisons of proportions, means and medians were performed using the Chi-square test, Student t, and Wilcoxon rank-sum tests respectively. Separate subgroup analyses were performed for both pneumonia and non-pneumonia populations. We assessed the discrimination of the ROX index using receiving operating characteristic (ROC) curves. Using parameters at HFNC initiation and one hour later, the Youden index method was used to determine the optimal cut-point of the ROX index for HFNC failure. Statistical significance was taken as a two-tailed P<0.05, and analyses were performed with Stata 15.0 (College Station, TX).

## Results

### Patient characteristics and outcomes

**Fig 1:**
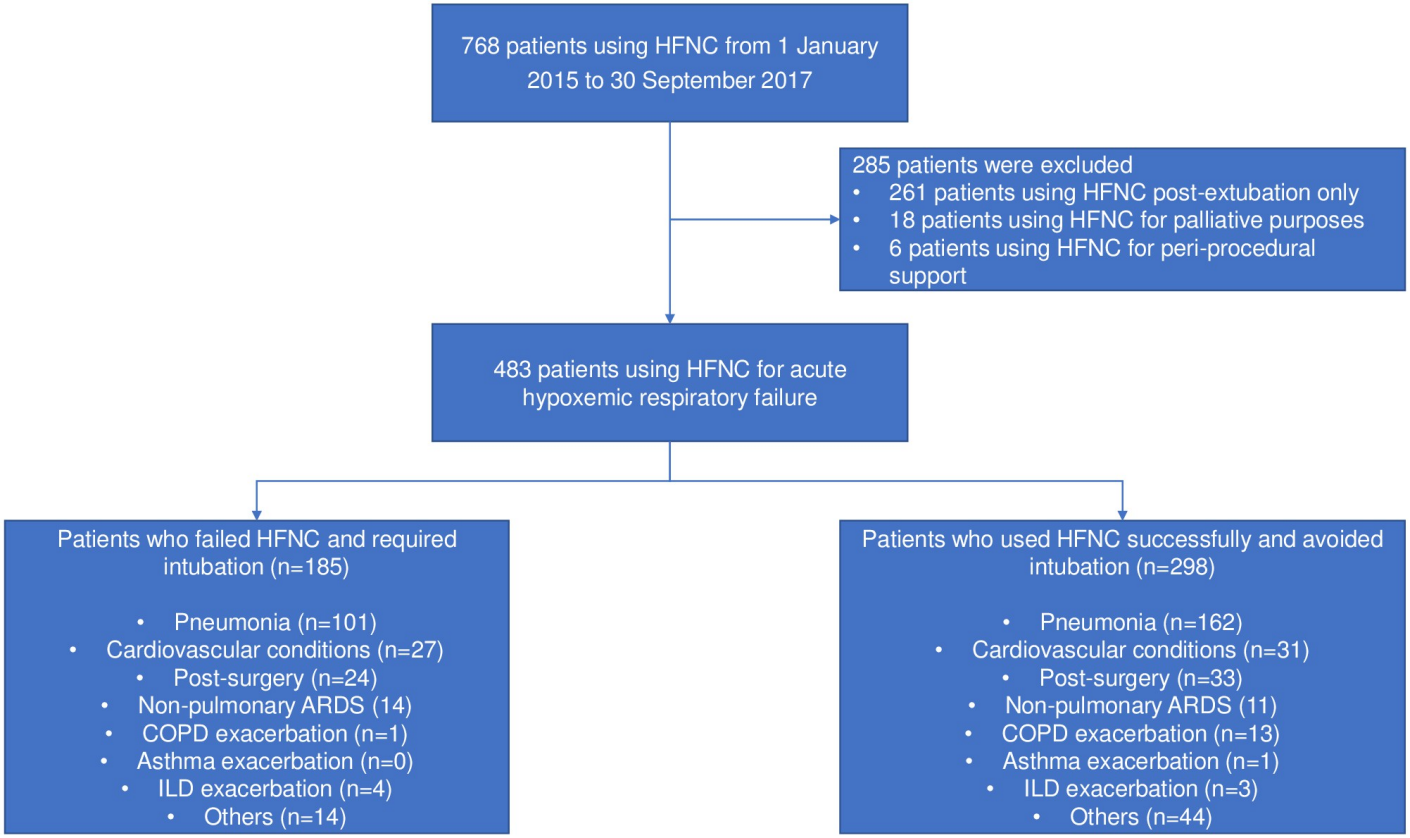
Enrolment and outcomes.

Eight ICUs participated in this study. 483 patients required HFNC for AHRF, of whom 45 patients also had concomitant hypercapnia. 263 patients (54%) had a primary diagnosis of pneumonia (Fig 1). The remaining patients who received HFNC had cardiovascular conditions (n = 58), utilized HFNC post-surgery (n = 57), and for non-pulmonary acute respiratory distress syndrome (n = 25), COPD (n = 14), interstitial lung disease (n = 7) or asthma exacerbations (n = 1). 185 patients (38.3%) failed HFNC. Baseline demographics were mostly similar between both patients with pneumonia and non-pneumonia conditions. Patients with non-pneumonia conditions were more likely to have ischemic heart disease and less likely to be admitted from the operating theatre (Table 1).

**Table 1:** Baseline characteristics of patients with hypoxemic respiratory failure.

	All patients (n = 483)	Patients with pneumonia (n = 263)	Patients with non-pneumonia conditions (n = 220)	p value
Median Age, (Years)	64 (55–74)	65 (55–74)	63 (56–73)	0.689
Male	345 (71.4%)	190 (72.2%)	155 (70.5%)	0.687
Median BMI (kg/m^2^)	22.9 (20.0–26.7)	22.8 (19.6–26.0)	22.9 (21.2–27.0)	0.074
Median APACHE II	19 (13–25)	19 (13–25)	20 (13–25)	0.633
Vasopressor use	94 (19.5%)	43 (16.4%)	51 (23.2%)	0.065
Smoking status				
Smoker	86 (17.8%)	40 (15.2%)	46 (20.9%)	0.271
Ex-smoker	335 (69.4%)	188 (71.5%)	147 (66.8%)	
Non-smoker	62 (12.8%)	35 (13.3%)	27 (12.3%)	
Admission source				
Emergency Department	207 (42.9%)	83 (37.7%)	124 (47.2%)	<0.001*
General Ward	233 (48.2%)	102 (46.4%)	131 (49.8%)	
Operating Theatre	43 (8.9%)	35 (15.9%)	8 (3.0%)	
Comorbidities				
Hypertension	282 (58.4%)	149 (56.65%)	133 (60.5%)	0.406
Diabetes	181 (37.5%)	100 (38.0%)	81 (36.8%)	0.850
Immunosuppression	133 (27.5%)	67 (25.5%)	66 (30.0%)	0.306
Chronic kidney disease	104 (21.5%)	59 (22.4%)	45 (20.5%)	0.657
Ischemic Heart Disease	103 (21.3%)	40 (15.2%)	63 (28.6%)	0.001*
COPD	34 (7.0%)	14 (5.3%)	20 (9.1%)	0.112
Stroke	32 (6.6%)	17 (6.5%)	15 (6.8%)	1.000
Asthma	28 (5.8%)	12 (4.6%)	16 (7.3%)	0.242
Liver cirrhosis	15 (3.1%)	7 (2.7%)	8 (3.6%)	0.604
Bronchiectasis	11 (2.3%)	8 (3.0%)	3 (1.4%)	0.359
Interstitial lung disease	9 (1.9%)	4 (1.5%)	5 (2.3%)	0.738
Outcomes				
Median duration of HFNC use (Hrs)	23 (9–49)	23 (10–53)	21 (9–46)	0.614
Median ICU length of stay (days)	6 (3–10)	6 (3–11)	6 (3–10)	0.503
Median hospital length of stay (days)	18 (10–35)	15 (9–29)	21 (11–41)	0.023*
ICU mortality	71 (14.7%)	47 (17.9%)	24 (10.9%)	0.039*
Hospital mortality	120 (24.8%)	78 (29.7%)	42 (19.1%)	0.008*

* represents those parameters that are statistically significant.

** BMI = Body mass index, APACHE II = Acute Physiology and Chronic Health Evaluation II, ED = Emergency Department, COPD = Chronic obstructive pulmonary disease.

### Performance of the ROX index among pneumonia patients only

Out of 263 patients with pneumonia, 101 (38.4%) required intubation. Among those who failed HFNC, they were more tachypneic [27 (23–33) breaths/min vs 24 (20–28) breaths/min, p = 0.006], tachycardic [106 (89–118) beats/min vs 95 (83–107) beats/min, p<0.001] and were initiated on HFNC at lower SF ratio [176 (97–243) vs 208 (115–257), p = 0.006] immediately prior to HFNC initiation. None of the other non-respiratory parameters were predictive of HFNC failure. These differences persisted one hour after HFNC initiation (Table 2). Those who failed HFNC already required higher FiO_2_ one hour of HFNC initiation [60% (50%-70%) vs 50% (40%-60%), p<0.001]. These results remained consistent when patients with concomitant hypercapnia were excluded (S1 Table).

**Table 2:** Parameters of patients with hypoxemic respiratory failure at various stages of HFNC use.

Parameters	Patients with pneumonia only (n = 263)			Patients with non-pneumonia conditions only (n = 220)		
	Patients who failed HFNC and required intubation (n = 101)	Patients who used HFNC successfully and avoided intubation (n = 162)	p value	Patients who failed HFNC and required intubation (n = 84)	Patients who used HFNC successfully and avoided intubation (n = 136)	p value
Clinical and serological parameters (Median, Inter-quartile range)						
Immediately prior to HFNC initiation						
Pre-HFNC BIPAP use (%)	18 (17.8)	33 (20.5)	0.634	20 (23.8)	41 (30.2)	0.354
Pre-HFNC CPAP use (%)	11 (10.9)	26 (16.2)	0.277	13 (15.5)	23 (16.9)	0.853
Respiratory rate (breaths/min)	27 (23–33)	24 (20–28)	0.006*	25 (21–29)	24 (19–29)	0.417
FiO2 (%)	50 (40–100)	50 (36–80)	0.007*	45 (36–50)	40 (35–50)	0.516
SpO2 (%)	95 (92–98)	95 (92–98)	0.553	95 (91–98)	95 (91–97)	0.775
SF ratio	176 (97–243)	208 (115–257)	0.006*	200 (170–257)	238 (185–277)	0.035*
ROX index	5.93 (3.93–8.97)	8.18 (5.00–11.55)	<0.001*	7.92 (6.06–10.78)	10.02 (7.23–13.04)	0.021*
PaCO2 (mmHg)	33 (29–40)	34 (30–37)	0.356	35 (31–39)	35 (29–39)	0.426
Serum HCO3 (mmol/l)	22.6 (20.0–26.4)	22.5 (19.3–26.0)	0.150	22.5 (19.2–26.6)	23.0 (20.2–26.2)	0.064
pH	7.44 (7.41–7.48)	7.43 (7.39–7.48)	0.208	7.43 (7.37–7.48)	7.41 (7.38–7.46)	0.617
Heart rate (bpm)	106 (89–118)	95 (83–107)	<0.001*	96 (83–113)	100 (81–114)	0.960
Systolic blood pressure (mmHg)	136 (115–157)	128 (111–153)	0.256	121 (101–141)	126 (107–145)	0.351
Diastolic blood pressure (mmHg)	75 (63–85)	72 (61–83)	0.661	66 (52–78)	70 (60–81)	0.072
Median GCS	15 (15–15)	15 (15–15)	0.175	15 (14–15)	15 (15–15)	0.436
1hr after HFNC administered						
Respiratory rate (breaths/min)	26 (23–31)	23 (19–30)	0.004*	24 (19–28)	22 (18–27)	0.338
Flow (L/min)	50 (50–60)	50 (40–50)	0.605	50 (45–60)	50 (40–60)	0.783
FiO2 (%)	60 (50–70)	50 (40–60)	<0.001*	50 (40–60)	40 (35–50)	0.001*
SpO2 (%)	96 (93–98)	96 (94–99)	0.436	95 (93–97)	96 (94–98)	0.877
SF ratio	164 (134–194)	190 (158–232)	<0.001*	196 (165–240)	240 (186–271)	0.001*
ROX index	6.74 (4.53–8.70)	8.56 (6.74–11.19)	<0.001*	8.03 (6.58–11.06)	10.35 (7.48–14.00)	0.001*
PaCO2 (mmHg)	33 (29–38)	34 (29–38)	0.823	34 (30–39)	33 (28–38)	0.814
Serum HCO3 (mmol/l)	23.1 (20.0–27.0)	23.0 (20.7–26.0)	0.223	23.8 (20.3–26.0)	22.0 (20.0–24.5)	0.259
pH	7.45 (7.40–7.49)	7.44 (7.40–7.47)	0.616	7.44 (7.40–7.50)	7.44 (7.40–7.47)	0.440
Heart rate (bpm)	102 (86–118)	92 (82–103)	<0.001*	98 (83–112)	96 (81–106)	0.289
Systolic blood pressure (mmHg)	133 (116–150)	129 (111–149)	0.727	119 (107–137)	120 (106–137)	0.916
Diastolic blood pressure (mmHg)	69 (61–81)	69 (60–79)	0.809	68 (55–77)	67 (59–77)	0.564
Median GCS	15 (15–15)	15 (15–15)	0.682	15 (14–15)	15 (15–15)	0.389

* represents those parameters that are statistically significant.

The ROX index was more discriminatory among pneumonia patients one-hour post-initiation [AUC 0.71 (95% CI 0.64–0.79)] as compared to upon HFNC initiation [AUC 0.65 (0.57–0.72)]. A ROX index <4.58 at HFNC initiation and <6.06 one-hour post-initiation predicted HFNC failure with 80% specificity (Table 3 and S1 Fig).

**Table 3:** Comparison of cut-off points and ROC curves between ROX index at initiation and after 1 hour of HFNC.

Index/ score	Cut-off point	Sensitivity at cut-off point	Specificity at cut-off point	Positive predictive value at cut-off point	Negative predictive value at cut-off point	Positive likelihood ratio at cut-off point	Negative likelihood ratio at cut-off point	AUC (95% CI)
All AHRF patients								
ROX	6.93 (Youden)	0.52	0.69	0.51	0.7	1.67	0.7	0.62 (0.57–0.68)
	5.18	0.31	0.8	0.49	0.65	1.55	0.86	
	3.84	0.16	0.91	0.52	0.64	1.78	0.92	
ROX at 1h	7.96 (Youden)	0.59	0.65	0.51	0.72	1.69	0.63	0.65 (0.60–0.71)
	6.46	0.37	0.8	0.53	0.67	1.85	0.79	
	4.94	0.22	0.91	0.6	0.66	2.44	0.86	
AHRF patients with pneumonia only								
ROX	6.56 (Youden)	0.59	0.67	0.52	0.73	1.79	0.61	0.65 (0.57–0.72)
	4.58	0.41	0.81	0.57	0.69	2.16	0.73	
	3.62	0.21	0.91	0.59	0.65	2.33	0.87	
ROX at 1h	7.18 (Youden)	0.65	0.68	0.55	0.76	2.03	0.51	0.71 (0.64–0.79)
	6.06	0.51	0.8	0.61	0.73	2.55	0.61	
	4.64	0.33	0.91	0.69	0.69	3.67	0.74	
AHRF patients not due to non-pneumonia conditions								
ROX	7.59 (Youden)	0.53	0.72	0.54	0.71	1.89	0.65	0.62 (0.55–0.69)
	6.24	0.33	0.80	0.51	0.66	1.65	0.84	
	4.36	0.14	0.90	0.46	0.63	1.40	0.96	
ROX at 1h	11.54 (Youden)	0.82	0.42	0.47	0.79	1.41	0.43	0.63 (0.56–0.70)
	6.89	0.34	0.80	0.51	0.66	1.79	0.83	
	5.44	0.13	0.91	0.45	0.63	1.44	0.96	

AUC: Area under the receiver operating characteristic curve.

CI: Confidence interval.

### Performance of the ROX index among non-pneumonia patients only

Among 220 patients with non-pneumonia AHRF, 84 (38.2%) failed HFNC. Patients who failed HFNC predominantly had lower median SF ratio compared to those who used HFNC successfully [200 (170–257) vs 238 (185–277), p = 0.035] immediately prior to HFNC initiation. Unlike pneumonia patients, patients who failed HFNC were not more tachypneic or tachycardic. One-hour after HFNC administration, these clinical characteristics still persisted. Again, higher FiO_2_ was already required among those who failed HFNC [50% (40%-60%) vs 40% (35%-50%)], p = 0.001] (Table 2). These results remained consistent when patients with concomitant hypercapnia were excluded (S1 Table).

The ROX index still showed moderate discrimination at HFNC initiation [AUC 0.62 (95% CI 0.55–0.69)] and one hour later [AUC 0.63 (95% CI 0.56–0.70)]. Correspondingly, a ROX index of <6.24 at HFNC initiation and <6.89 one-hour later predicted HFNC failure (Table 3 and S1 Fig).

## Discussion

Among AHRF patients with pneumonia, the ROX index demonstrated moderate discriminatory power to predict HFNC failure, with greater discrimination one-hour post-initiation compared to when HFNC was initiated. In a similar fashion, the ROX index was also validated as moderately discriminating among non-pneumonia AHRF patients at similar time points.

Roca and colleagues originally validated the ROX index in patients with pneumonia [16,17]. However, at least 20% of all ICU patients with AHRF suffer from non-pneumonia-related conditions [21]. In some subgroups such as immunocompromised patients, the etiology of AHRF is even more varied [20,22–25], and in ED, HFNC may be initiated before the underlying etiology of AHRF is established [9]. Since delayed intubation can increase mortality regardless of the cause of AHRF [7], it is important to understand the performance of the ROX index among patients with non-pneumonia-related conditions. Currently, there are few studies investigating HFNC use among this population and they mainly focused on demonstrating physiological benefits [2,7,26–28]. In contrast, our study focused on understanding the performance of the ROX index among a more diverse HFNC population.

In addition, as the ROX index was originally validated at 2, 6 and 12 hours [16,17], we explored its ability to discriminate patients likely to fail HFNC at earlier time points. This would facilitate early pre-emptive intubation before further deterioration has occurred [15]. We demonstrated that at one-hour post-HFNC initiation, the AUROC of the ROX index for predicting HFNC failure was 0.71 and 0.63 for pneumonia and non-pneumonia patients respectively. Therefore, we have shown that the ROX index calculated immediately prior to HFNC initiation and one-hour post-initiation can help identify those likely to fail. Since HFNC is increasingly initiated while patients are still in ED, predicting HFNC failure at earlier time points can guide the development of practical workflows for close monitoring, especially in settings where staffing resources for close patient monitoring is scarce, or transfer time from ED to ICU is prolonged. Identifying patients most likely to fail HFNC may facilitate earlier intubation decisions and increase safety during patient transport [29].

It is important to highlight that the ROX index is a screening tool with only moderate discriminatory power. In the original study, Roca and colleagues report AUROC values at 2 hours of 0.679, 6 hours of 0.703 and 12 hours of 0.752 [17]. We report similar values in our study (Table 3). To maximize the screening tool potential of the ROX index, the values adopted favoured higher specificity and negative predictive values, so as to reduce false positives. To illustrate, a ROX index of <6.06 (sensitivity 51%, specificity 80%) among pneumonia patients one-hour post-HFNC initiation would have led to an additional 46 patients being intubated. Importantly, our study also highlights the importance of serial measurements of the ROX index given the dynamic evolution of the disease course among these patients. Depending on the study population, the exact ROX index value predicting HFNC failure may differ. This is illustrated with the studies by Roca and Goh separately depicting different values at the same time points at 2 (<2.85 vs <6.55), 6 (<3.47 vs <6.60) and 12 (<3.85 vs <6.55) hours [17,18].

Our study has several strengths. It is a practical real-world evaluation. Our study population was large, from multiple centers and included AHRF patients with multiple etiologies. This allowed us to validate the ROX index at earlier time points and among non-pneumonia patients. To date, this is the largest study population investigating HFNC failure among non-pneumonia patients. However, we acknowledge several limitations in our study. Firstly, it was conducted retrospectively, and in the absence of a standardized protocol, we were unable to study the ROX index at other time points, in particular at later time points, which could be a subject for future studies. Secondly, the lack of a standardized protocol for HFNC practice could have led to biases from individual physician practice patterns and thresholds for determining HFNC failure. Thirdly, as with all retrospective studies, some missing data is inevitable, with the majority related to arterial blood gas findings one-hour post-HFNC initiation. Other studies have already established that these parameters do not predict HFNC failure [16,18,19]. Otherwise, all other parameters were missing <10% of the total data, thus mitigating the bias risk (S1 Table). Fourthly, determination of HFNC failure was clinically-driven rather than protocolized, and some variation of HFNC failure thresholds existed across centers (S3 and S4 Tables). Importantly, our HFNC failure rate of 38.3% is also similar to that of the FLORALI study [7]. Finally, in the absence of randomization, we are unable to account for the effect of unmeasured variables that may affect the ROX index.

## Conclusion

There is moderate discriminatory power of the ROX index to predict HFNC failure among both pneumonia and non-pneumonia patients with AHRF upon HFNC initiation and one-hour post-initiation.

## Supporting information

S1 Fig ROC Curves of the ROX index prior and one-hour post-HFNC initiation among both pneumonia and non-pneumonia patients. (PPTX)

S1 Table Parameters of patients with only hypoxemic respiratory failure at various stages of HFNC use. (DOCX)

S2 Table Missing data upon HFNC initiation and one-hour post HFNC initiation. (DOCX)

S3 Table Median values of parameters of patients at each site who failed HFNC at the point of intubation. (DOCX)

S4 Table Parameters of patients with pneumonia and non-pneumonia conditions who failed HFNC at the point of intubation. (DOCX)

S1 Dataset Key to dataset. (XLSX)

S2 Dataset Minimal anonymized data set. (XLSX)

## Abbreviations

ABG: Arterial Blood Gas
AHRF: Acute hypoxemic respiratory failure
COT: Conventional oxygen therapy
COVID-19: Coronavirus disease 2019
ED: Emergency Department
GCS: Glasgow Coma Scale
HFNC: High flow nasal cannula
ICU: Intensive Care Unit
NIV: Non-invasive ventilation
