# Correction: Expanding the utility of the ROX index among patients with acute hypoxemic respiratory failure

**DOI:** 10.1371/journal.pone.0282136

**Published:** 2023-02-16

**Authors:** Andrew Li, Matthew Edward Cove, Jason Phua, Ser Hon Puah, Vicky Ng, Amit Kansal, Qiao Li Tan, Juliet Tolentino Sahagun, Juvel Taculod, Addy Yong-Hui Tan, Amartya Mukhopadhyay, Chee Kiang Tay, Kollengode Ramanathan, Yew Woon Chia, Duu Wen Sewa, Meiying Chew, Sennen J. W. Lew, Shirley Goh, Shekhar Dhanvijay, Jonathan Jit-Ern Tan, Kay Choong See, on behalf of SICM-NICER Group

The last author’s name is incorrect. The correct name is: Kay Choong See. The author’s initials are also indexed incorrectly in PubMed. The correct initials are: See KC.
