# Supplementary material for: Expanding the utility of the ROX index among patients with acute hypoxemic respiratory failure
Source: PLoS One. 2022 Apr 26;17(4):e0261234. doi: 10.1371/journal.pone.0261234 (PMC9041854; doi:10.1371/journal.pone.0261234)
Supplement: S1 Fig — (PPTX) [file pone.0261234.s001.pptx]

## Slide 1
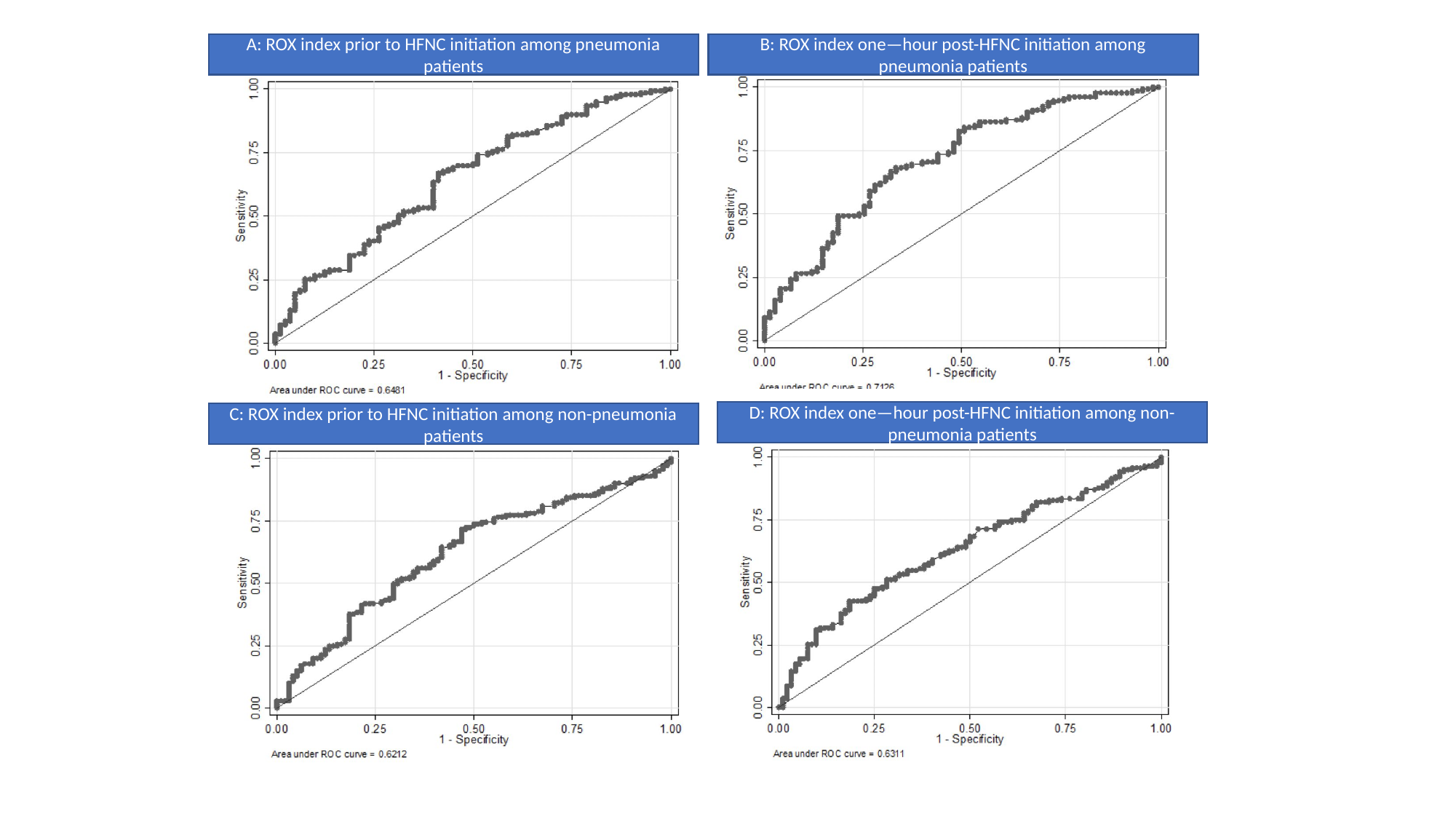

B: ROX index one—hour post-HFNC initiation among pneumonia patients
A: ROX index prior to HFNC initiation among pneumonia patients
D: ROX index one—hour post-HFNC initiation among non-pneumonia patients
C: ROX index prior to HFNC initiation among non-pneumonia patients
