# Supplementary material for: Expanding the utility of the ROX index among patients with acute hypoxemic respiratory failure
Source: PLoS One. 2022 Apr 26;17(4):e0261234. doi: 10.1371/journal.pone.0261234 (PMC9041854; doi:10.1371/journal.pone.0261234)
Supplement: S1 Table — (DOCX) [file pone.0261234.s002.docx]

| Supplementary table 1: Parameters of patients with only hypoxemic respiratory failure at various stages of HFNC use | | | | | | | | | |
| --- | --- | --- | --- | --- | --- | --- | --- | --- | --- |
| Parameters | All patients with hypoxemic respiratory failure (n=438) | | | Patients with pneumonia only (n=240) | | | Patients with non-pneumonia conditions only (n=198) | | |
|  | Patients who failed HFNC and required intubation (n=167) | Patients who used HFNC successfully and avoided intubation (n=271) | p value | Patients who failed HFNC and required intubation (n=91) | Patients who used HFNC successfully and avoided intubation (n=149) | p value | Patients who failed HFNC and required intubation (n=76) | Patients who used HFNC successfully and avoided intubation (n=122) | p value |
| Clinical and serological parameters (Median, Inter-quartile range) | | | | | | | | | |
| Immediately prior to HFNC initiation | | | | | | | | | |
| Pre-HFNC BIPAP use (%) | 30 (18.0%) | 64 (23.6%) | 0.187 | 14 (15.4%) | 31 (20.8%) | 0.311 | 16 (21.1%) | 33 (27.1%) | 0.399 |
| Pre-HFNC CPAP use (%) | 23 (13.8%) | 47 (17.3%) | 0.349 | 10 (11.0%) | 26 (17.4%) | 0.195 | 13 (17.1%) | 21 (17.2%) | 1.000 |
| Respiratory rate (breaths/min) | 26 (22-31) | 24 (20-29) | 0.010* | 28 (23-33) | 24 (20-28) | 0.001* | 25 (21-30) | 25 (20-30) | 0.764 |
| FiO2 (%) | 50 (36-80) | 42 (35-50) | 0.006* | 50 (40-100) | 50 (35-80) | 0.002* | 45 (36-50) | 40 (35-50) | 0.666 |
| SpO2 (%) | 95 (91-98) | 95 (92-97) | 0.819 | 95 (92-98) | 95 (92-98) | 0.426 | 95 (91-98) | 95 (92-97) | 0.663 |
| SF ratio | 186 (117-247) | 216 (173-266) | <0.001* | 170 (97-243) | 194 (121-258) | 0.002* | 200 (170-253) | 235 (184-274) | 0.041* |
| ROX index | 6.87 (4.33-9.81) | 9.00 (6.15-12.07) | <0.001* | 5.93 (3.73-8.80) | 8.32 (5.53-11.74) | <0.001* | 8.03(5.94-10.73) | 9.54 (7.13-12.35) | 0.045* |
| pCO2 (mmHg) | 33.0 (30.0-37.5) | 33.0 (29.0-37.0) | 0.423 | 32.0 (29.0-37.0) | 33.0 (30.0-36.0) | 0.423 | 34.0 (30.6-38.0) | 33.0 (29.0-38.0) | 0.402 |
| Serum HCO3 (mmol/l) | 22.2 (20.0-25.5) | 21.9 (19.0-24.9) | 0.043* | 22.0 (20.0-25.0) | 22.0 (19.0-25.0) | 0.277 | 22.9 (20.0-26.0) | 21.9 (19.0-24.4) | 0.094 |
| pH | 7.44 (7.39-7.48) | 7.43 (7.39-7.47) | 0.378 | 7.44 (7.40-7.47) | 7.43 (7.40-7.48) | 0.485 | 7.43 (7.38-7.48) | 7.42 (7.38-7.47) | 0.553 |
| Heart rate (bpm) | 101 (87-116) | 98 (83-109) | 0.034* | 106 (90-117) | 96 (84-107) | <0.001* | 95 (81-112) | 101 (83-116) | 0.478 |
| Systolic blood pressure (mmHg) | 126 (109-149) | 125 (108-147) | 0.737 | 135 (114-155) | 125 (110-152) | 0.258 | 121 (100-142) | 125 (107-145) | 0.468 |
| Diastolic blood pressure (mmHg) | 70 (55-81) | 71 (60-82) | 0.327 | 74 (63-84) | 72 (60-82) | 0.955 | 66 (52-78) | 70 (60-81) | 0.111 |
| Median GCS | 15 (15-15) | 15 (15-15) | 0.190 | 15 (15-15) | 15 (15-15) | 0.476 | 15 (15-15) | 15 (15-15) | 0.302 |
| 1hr after HFNC administered | | | | | | | | | |
| Respiratory rate (breaths/min) | 25 (21-30) | 23 (19-27) | 0.005* | 27 (21-31) | 24 (19-28) | 0.003* | 23 (19-30) | 22 (18-27) | 0.392 |
| Flow (L/min) | 50 (50-60) | 50 (40-60) | 0.750 | 50 (50-60) | 50 (45-60) | 0.483 | 50 (40-60) | 50 (40-60) | 0.703 |
| FiO2 (%) | 55 (45-70) | 45 (40-50) | <0.001* | 60 (50-70) | 50 (40-50) | <0.001* | 50 (40-60) | 40 (35-50) | 0.001* |
| SpO2 (%) | 96 (93-99) | 96 (94-98) | 0.753 | 95 (93-98) | 95 (93-97) | 0.619 | 96 (95-99) | 97 (94-98) | 0.925 |
| SF ratio | 166 (137-200) | 200 (182-250) | <0.001* | 163 (134-192) | 196 (167-240) | <0.001* | 188 (158-230) | 238 (188-269) | 0.001* |
| ROX index | 7.06 (5.05-9.68) | 9.33 (7.00-12.48) | <0.001* | 6.33 (4.44-8.70) | 8.59 (6.76-11.1) | <0.001* | 7.90 (6.20-11.06) | 10.33 (7.48-13.6) | 0.001* |
| pCO2 (mmHg) | 32.0 (29.0-37.0) | 32.0 (29.0-36.3) | 0.759 | 31.9 (29.0-37.0) | 33.0 (30.0-36.0) | 0.693 | 32.5 (28.0-36.4) | 32.0 (28.0-37.0) | 0.980 |
| Serum HCO3 (mmol/l) | 23.1 (19.8-25.7) | 22.4 (20.0-24.6) | 0.204 | 22.6 (19.8-25.6) | 22.9 (20.0-25.3) | 0.409 | 23.5 (20.0-26.0) | 21.9 (19.6-24.3) | 0.211 |
| pH | 7.44 (7.41-7.50) | 7.44 (7.40-7.48) | 0.516 | 7.44 (7.40-7.49) | 7.44 (7.41-7.48) | 0.847 | 7.44 (7.41-7.51) | 7.44 (7.40-7.47) | 0.227 |
| Heart rate (bpm) | 100 (84-115) | 94 (82-105) | 0.002* | 100 (84-115) | 94 (82-105) | <0.001* | 104 (86-117) | 87 (79-94) | 0.752 |
| Systolic blood pressure (mmHg) | 127 (113-146) | 124 (109-144) | 0.503 | 132 (115-151) | 128 (111-148) | 0.719 | 122 (107-140) | 119 (105-135) | 0.526 |
| Diastolic blood pressure (mmHg) | 69 (57-80) | 68 (59-78) | 0.656 | 69 (59-80) | 69 (60-79) | 0.741 | 69 (55-78) | 67 (59-77) | 0.764 |
| Median GCS | 15 (15-15) | 15 (15-15) | 0.227 | 15 (15-15) | 15 (15-15) | 0.616 | 15 (15-15) | 15 (15-15) | 0.253 |
|  |  |  |  |  |  |  |  |  |  |
|  |  |  |  |  |  |  |  |  |  |

* represents those parameters that are statistically significant
