# Supplementary material for: Expanding the utility of the ROX index among patients with acute hypoxemic respiratory failure
Source: PLoS One. 2022 Apr 26;17(4):e0261234. doi: 10.1371/journal.pone.0261234 (PMC9041854; doi:10.1371/journal.pone.0261234)
Supplement: S2 Table — (DOCX) [file pone.0261234.s003.docx]

| Supplementary table 2: Missing data upon HFNC initiation and one-hour post HFNC initiation | |
| --- | --- |
| Parameters | Number of patients with missing data^a^ |
| Baseline demographics | |
| BMI | 47 (9.7%) |
| APACHE II | 11 (2.3%) |
| Parameters immediately prior to HFNC initiation | |
| pH | 62 (12.8%) |
| PaCO2 | 62 (12.8%) |
| Serum HCO3 | 62 (12.8%) |
| FiO2 | 16 (3.3%) |
| SpO2 | 8 (6.6%) |
| SF ratio | 22 (4.6%) |
| Respiratory rate | 14 (2.9%) |
| ROX index | 25 (5.2%) |
| Heart rate | 16 (3.3%) |
| Systolic blood pressure | 64 (13.3%) |
| Diastolic blood pressure | 64 (13.3%) |
| GCS | 29 (6.0%) |
| Parameters 1-hour post-HFNC initiation | |
| pH | 212 (43.9%) |
| PaCO2 | 212 (43.9%) |
| Serum HCO3 | 212 (43.9%) |
| FiO2 | 46 (9.5%) |
| SpO2 | 42 (8.7%) |
| SF ratio | 44 (9.1%) |
| Respiratory rate | 44 (9.1%) |
| ROX index | 45 (9.3%) |
| Heart rate | 42 (9.7%) |
| Systolic blood pressure | 102 (21.1%) |
| Diastolic blood pressure | 102 (21.1%) |
| GCS | 66 (13.7%) |
| Outcomes | |
| ICU length of stay | 2 (0.4%) |
| Hospital length of stay | 1 (0.2%) |

^a^ Numbers refer to patients with selected missing fields in case report forms which could not be rectified despite attempts to contact ICU representatives for clarification.

* BMI = Body mass index, APACHE II = Acute Physiology and Chronic Health Evaluation II, ICU = Intensive care unit
