# Supplementary material for: Expanding the utility of the ROX index among patients with acute hypoxemic respiratory failure
Source: PLoS One. 2022 Apr 26;17(4):e0261234. doi: 10.1371/journal.pone.0261234 (PMC9041854; doi:10.1371/journal.pone.0261234)
Supplement: S3 Table — (DOCX) [file pone.0261234.s004.docx]

| Supplementary table 3: Median values of parameters of patients at each site who failed HFNC at the point of intubation | | | | | | | | |  |
| --- | --- | --- | --- | --- | --- | --- | --- | --- | --- |
| Parameters | Site 1 (n=30) | Site 2 (n=50) | Site 3 (n=3) | Site 4 (n=30) | Site 5 (n=4) | Site 6 (n=21) | Site 7 (n=27) | Site 8 (n=22) | |
| Pneumonia patients (No.) | 26 | 31 | 0 | 18 | 4 | 8 | 14 | 0 | |
| Respiratory rate (breaths/min) | 27 (23-34) | 24 (21-27) | ND | 26 (22-30) | ND | 27 (22-32) | 30 (25-36) | ND | |
| Flow (L/min) | 60 (50-60) | 50 (40-60) | ND | 55 (50-60) | ND | 60 (55-60) | 55 (45-60) | ND | |
| FiO2 (%) | 70 (60-80) | 55 (50-70) | ND | 60 (50-75) | ND | 55 (45-75) | 45 (35-60) | ND | |
| SpO2 (%) | 92 (92-95) | 95 (92-97) | ND | 95 (94-97) | ND | 92 (84-95) | 95 (92-99) | ND | |
| SF ratio | 136 (102-157) | 176 (129-226) | ND | 150 (129-189) | ND | 158 (120-238) | 200 (158-266) | ND | |
| ROX index | 4.52 (3.61-6.74) | 6.67 (5.79-8.52) | ND | 5.78 (5.22-7.37) | ND | 5.88 (4.59-8.33) | 6.90 (4.71-9.52) | ND | |
| PaCO2 (mmHg) | 36.0 (27.0-39.0) | 38.0 (3.0-41.0) | ND | 33.0 (30.0-39.9) | ND | 33.5 (28.5-44.5) | 31.0 (24.0-33.0) | ND | |
| Serum HCO3 (mmol/l) | 22.0 (18.0-25.0) | 23.5 (22.1-29.3) | ND | 26.2 (21.3-29.7) | ND | 20.5 (17.5-24.5) | 20.8 (17.7-23.3) | ND | |
| pH | 7.40 (7.36-7.45) | 7.48 (7.47-7.50) | ND | 7.47 (7.41-7.50) | ND | 7.35 (7.32-7.45) | 7.46 (7.41-7.48) | ND | |
| Heart rate (bpm) | 106 (78-124) | 101 (89-123) | ND | 103 (90-132) | ND | 121 (120-126) | 110 (82-128) | ND | |
| Systolic blood pressure (mmHg) | 120 (114-161) | 128 (120-139) | ND | 133 (117-148) | ND | 126 (101-154) | 110 (103-113) | ND | |
| Diastolic blood pressure (mmHg) | 75 (56-88) | 78 (64-81) | ND | 76 (60-92) | ND | 72 (57-84) | 68 (50-74) | ND | |
| Median GCS | 15 (15-15) | 15 (15-15) | ND | 15 (11-15) | ND | 15 (14-15) | 13 (8-15) | ND | |
| Non-pneumonia patients (No.) | 4 | 19 | 3 | 12 | 0 | 17 | 7 | 22 | |
| Respiratory rate (breaths/min) | 33 (28-39) | 22 (20-29) | 27 (16-29) | 30 (19-30) | ND | 24 (18-31) | 21 (17-37) | 25 (18-31) | |
| Flow (L/min) | 60 (60-60) | 60 (50-60) | 60 (60-60) | 50 (40-50) | ND | 50 (50-60) | 45 (40-60) | 50 (50-50) | |
| FiO2 (%) | 65 (50-85) | 60 (50-70) | 45 (40-50) | 60 (50-60) | ND | 50 (4060) | 60 (50-70) | 50 (40-60) | |
| SpO2 (%) | 91 (84-96) | 94 (93-96) | 94 (92-95) | 94 (91-97) | ND | 93 (88-96) | 94 (87-100) | 94 (89-98) | |
| SF ratio | 136 (117-195) | 170 (155-190) | 213 (190-230) | 178 (153-200) | ND | 174 (148-208) | 162 (134-170) | 168 (150-243) | |
| ROX index | 4.30 (3.346-5.87) | 6.65 (5.60-8.36) | 8.52 (6.55-13.3) | 5.93 (5.20-10.11) | ND | 7.50 (5.51-9.35) | 7.38 (5.23-9.8) | 8.10 (4.68-10.96) | |
| PaCO2 (mmHg) | 32.0 (30.0-37.5) | 38.0 (37.0-39.0) | 450 (29.0-54.0) | 31.8 (27.9-38.0) | ND | 37.0 (30.0-53.0) | ND | 32.4 (29.0-34.4) | |
| Serum HCO3 (mmol/l) | 18.0 (15.0-21.5) | 23.8 (19.3-28.5) | 23.0 (21.0-30.0) | 23.0 (21.625.3) | ND | 24.0 (20.0-26.0) | ND | 24.7 (23.0-27.0) | |
| pH | 7.31 (7.31-7.37) | 7.43 (7.28-7.49) | 7.35 (7.34-7.47) | 7.47 (7.42-7.53) | ND | 7.38 (7.36-7.43) | ND | 7.47 (.44-7.52) | |
| Heart rate (bpm) | 127 (90-158) | 98 (90-113) | 103 (88-118) | 95 (78-114) | ND | 86 (77-102) | 114 (7-118) | 88 (86-100) | |
| Systolic blood pressure (mmHg) | 126 (79-151) | 130 (124-147) | 137 (112-147) | 122 (87-150) | ND | 123 (115-142) | 107 (84-128) | 118 (101-134) | |
| Diastolic blood pressure (mmHg) | 64 (53-78) | 86 (74-94) | 79 (61-82) | 52 (48-60) | ND | 68 (61-78) | 70 (49-85) | 63 (49-84) | |
| Median GCS | 15 (15-15) | 15 (15-15) | 10 (9-10) | 15 (11-15) | ND | 14 (12-15) | 15 (14-15) | 15 (15-15) | |

Abbreviation: SF ratio, SpO_2_:FiO_2_ ratio; GCS, Glasgow coma scale; ND, No data;
