# Supplementary material for: Expanding the utility of the ROX index among patients with acute hypoxemic respiratory failure
Source: PLoS One. 2022 Apr 26;17(4):e0261234. doi: 10.1371/journal.pone.0261234 (PMC9041854; doi:10.1371/journal.pone.0261234)
Supplement: S4 Table — (DOCX) [file pone.0261234.s005.docx]

| Supplementary table 4: Parameters of patients with pneumonia and non-pneumonia conditions who failed HFNC at the point of intubation | | | |
| --- | --- | --- | --- |
| Parameters | All pneumonia patients (n=101) | All non-pneumonia patients (n=84) | P value |
| Respiratory rate (breaths/min) | 26 (22-30) | 25 (18-31) | 0.431 |
| Flow (L/min) | 60 (50-60) | 50 (50-60) | 0.195 |
| FiO2 (%) | 60 (50-80) | 55 (50-60) | 0.039* |
| SpO2 (%) | 94 (92-97) | 94 (90-96) | 0.429 |
| SF ratio | 154 (120-194) | 167 (150-200) | 0.071 |
| ROX index | 5.79 (4.56-7.77) | 7.28 (5.20-9.80) | 0.020* |
| PaCO2 (mmHg) | 34.0 (29.5-40.5) | 34.7 (29.6-39.9) | 0.422 |
| Serum HCO3 (mmol/l) | 22.8 (19.0-26.5) | 23.7 (21.0-27.0) | 0.649 |
| pH | 7.44 (7.36-7.48) | 7.43 (7.35-7.48) | 0.161 |
| Heart rate (bpm) | 108 (86-124) | 94 (83-109) | 0.012* |
| Systolic blood pressure (mmHg) | 128 (112-148) | 124 (110-145) | 0.501 |
| Diastolic blood pressure (mmHg) | 75 (60-84) | 70 (58-84) | 0.256 |
| Median GCS | 15 (14-15) | 15 (14-15) | 0.718 |
